# Impact of Sorting and Catch Media on Porcine Sperm Motility, Capacitation, and Viability

**DOI:** 10.1111/andr.70184

**Published:** 2026-01-28

**Authors:** Tyler Weide, Juan Steibel, Karl Kerns

**Affiliations:** ^1^ Interdepartmental Genetics and Genomics Iowa State University Ames Iowa USA; ^2^ Department of Animal Science Iowa State University Ames Iowa USA

**Keywords:** capacitation, cell sorting, mitochondria, motility, viability

## Abstract

**Background:**

Fluorescence‐activated cell sorting (FACS) has emerged as a powerful tool for selecting spermatozoa of a desired population of fluorescent biomarkers, offering potential applications in reproductive biology and agriculture. However, concerns remain regarding sorting‐induced physiological alterations that could compromise spermatozoa and/or their ability to capacitate.

**Objectives:**

Our objective was to determine whether catch media composition influences sperm recovery rate and physiological responses post‐sort, before and after in vitro capacitation (IVC).

**Materials and Methods:**

Five million spermatozoa per treatment were sorted and assessed immediately (0 h) and after 4 h of in vitro capacitation (4 h) using computer‐aided sperm analysis (CASA) and Image‐Based Flow Cytometry (IBFC). Treatments included six FACS‐sorted groups across three media types with BSA or PVA supplementation, and one unsorted control. Functional parameters, including motility, zinc efflux, oxidative stress, membrane remodeling, and mitochondrial activity, were quantitatively assessed.

**Results:**

We found that catch media without PVA or BSA had a near 10%–24% sperm recovery compared to 88%–92% with supplementation (*p *< 0.05). Immediately post‐sort, spermatozoa exhibited minimal alterations relative to controls; however, after 4 h IVC, sorted groups demonstrated significant shifts in zinc efflux (*p *< 0.05), increased plasma membrane remodeling (*p *< 0.05), and reduced mitochondrial activity (*p *< 0.05). While LTX‐based media preserved motility, differential responses in membrane integrity and zinc signature distribution were observed between treatments, suggesting that catch media can modulate capacitation outcomes. Notably, mitochondrial and membrane changes became more evident after capacitation incubation, indicating a delayed effect of sorting stress.

**Conclusion:**

These findings highlight the importance of optimizing catch media and incubation conditions to maintain post‐sorting sperm functionality and inform the refinement of FACS protocols for agricultural and assisted reproductive technologies. By minimizing sorting‐associated stress through media composition, sperm quality and fertilization potential may be better preserved.

## 1 Introduction

Sperm sorting is a widely used biotechnological approach in both livestock breeding and assisted reproductive technologies (ART). By separating X‐ and Y‐chromosome‐bearing spermatozoa, producers can optimize offspring sex ratios to increase herd genetics of economically desirable traits, such as increased milk production in dairy cattle or other terminal production characteristics in beef and pork [1, 2, 3, 4, 5]. Fluorescence‐Activated Cell Sorting (FACS) has revolutionized sperm selection by enabling high‐throughput identification and isolation based on DNA content, which was developed throughout the 1980s [6, 7, 8], and further optimized staining protocols were performed by Johnson et al. later in the decade [9, 10]. Sorting of sperm cells was first developed by Pinkel et al. in 1982 by separating sex populations in the vole *Microtus oregoni* [11], and the first reported use of H33342 DNA stain on live mammalian sperm cells was performed by Morrell et al. in 1988 [12]. From the 1980s and 1990s, there have been major efforts to optimize sorting of spermatozoa, such as lowering the fluidic pressure of the flow cytometer, which reduces the amount of stress placed on the sperm cell and increases the number of live spermatozoa in the sorted population [13]. However, while FACS has been well‐studied for its ability to separate spermatozoa by sex, its physiological effects on sperm function, particularly capacitation, remain poorly understood.

The process of sperm capacitation is a complex sequence of biochemical and physiological changes that prepares spermatozoa for fertilization, which was first identified in 1951 independently in rabbits [14] and mice [15]. Upon ejaculation, spermatozoa exist in a non‐capacitated state, with inhibitory factors bound to the plasma membrane to prevent premature activation. These decapacitating factors, including cholesterol and seminal plasma proteins, are gradually removed as sperm traverse the oviductal sperm reservoir, allowing for cholesterol [16, 17, 18, 19] and zinc efflux [20, 21], increased plasma membrane fluidity [17, 22, 23], and a series of signaling cascades. The intracellular pH increase, mediated by bicarbonate (HCO_3_^−^) and calcium (Ca^2^^+^) influx, plays a crucial role in activating protein kinase A (PKA) and tyrosine phosphorylation, leading to hyperactivation and ultimately the acrosome exocytosis across mammalian species [24, 25, 26, 27]. However, sorting procedures may inadvertently accelerate, delay, or impair sperm capacitation, influencing fertilization competency and sperm lifespan.

Although the impact of sorting on motility and viability has been previously investigated, how sorting affects capacitation kinetics and sperm function over time remains an open question. Sorting‐induced changes may result from membrane destabilization, oxidative stress from reactive oxygen species (ROS) generation, or disruption of ion channel regulation. The type of catch media used post‐sorting may further influence sperm capacitation by modulating the extent of membrane fluidity changes and oxidative stress experienced by sorted cells. Specifically, bovine serum albumin (BSA) is a well‐known capacitation inducer [28], acting as a cholesterol acceptor to promote capacitation‐like membrane changes. In contrast, polyvinyl alcohol (PVA) is an inert protein substitute that does not act as a cholesterol acceptor or facilitate cholesterol efflux, but can promote hyperactivation in boar spermatozoa when used in conjunction with cAMP analogs [29]. Additionally, the formulation of capacitation and non‐capacitation media can affect intracellular signaling, further influencing capacitation timing and efficiency post‐sorting.

Fluorescence‐based techniques, such as Image‐Based Flow Cytometry (IBFC), have been instrumental in analyzing sperm quality, viability, and capacitation status. These methods provide detailed assessments of membrane integrity, oxidative stress, acrosomal integrity, and intracellular signaling, offering a comprehensive approach to evaluating sperm function paired with the ability to observe morphology status. While capacitation is a pre‐requisite for successful fertilization, improper timing (e.g., early or delayed) or altered kinetics could negatively impact fertility outcomes. Despite its advantages in sperm selection, FACS introduces physical and biochemical stressors, including shear forces from fluidics, sorting pressure, and exposure to fluorescence dyes such as Hoechst 33342, which may impact sperm membrane stability, intracellular signaling, motility, and capacitation onset [30, 31, 32].

In both bovine and stallion spermatozoa, sex sorting is associated with elevated levels of tyrosine phosphorylation, which is linked to premature capacitation and modifications in glycocalyx structures in the plasma membrane [33, 34]. Furthermore, in cryopreserved bovine spermatozoa, sorted cells exhibited reduced motility, compromised plasma membrane integrity, and significantly lower acrosomal integrity compared to their non‐sorted counterparts [35]. Downstream from sperm quality, it has been reported that sex sorting bovine spermatozoa alters early embryonic development, significantly reduces blastocyst formation rate, and significantly increases the incidence of two‐ and four‐cell stage arrest [36]. These findings underscore the urgent need to refine sperm sorting protocols in order to preserve sperm function and viability, thereby enhancing the success of in vitro fertilization and improving the efficiency of sex selection in livestock production systems.

This study aims to evaluate the effects of FACS on sperm recovery, motility, and capacitation status by testing three different catch media: a long‐term extender, a short‐term extender, and a non‐capacitation medium supplemented with either 1% BSA or 0.1% PVA. These media were selected to determine whether protein supplementation (BSA vs. PVA) influences capacitation outcomes post‐sorting. We hypothesize that catch media will affect post‐sort recovery. We also hypothesize that sorting induces capacitation‐like changes in spermatozoa resulting from membrane perturbations, with media composition modulating these effects by influencing membrane permeability, intracellular signaling, and capacitation kinetics.

To test these hypotheses, we employed a high‐throughput, multiparametric approach, integrating computer‐aided sperm analysis (CASA) for motility assessment and IBFC to track capacitation biomarkers such as zinc efflux, membrane and acrosomal integrity, and oxidative stress markers. By analyzing these parameters across different sorting and media conditions, this study seeks to provide a comprehensive evaluation of the physiological effects of FACS on boar sperm function. Findings may contribute to the optimization of sperm sorting protocols for both livestock breeding and ART applications, ensuring that sorting procedures do not negatively impact capacitation timing, fertilization potential, or post‐sorting sperm lifespan.

## 2 Materials and Methods

### 2.1 Experimental Design

To evaluate the effects of FACS on sperm recovery, motility, and capacitation status, seven treatment groups were analyzed. These included an unsorted control and three experimental media: pNCM (porcine non‐capacitation media), short‐term extender (Beltsville Thaw Solution (BTS), Minitüb GmbH, Tiefenbach, Germany), and long‐term extender (AndroStar Premium, Minitüb GmbH, Tiefenbach, Germany). Each experimental medium was supplemented with either 1% BSA or 0.1% PVA in the catch media. These extenders were selected to evaluate differences in their ability to maintain sperm viability and functionality during sorting and capacitation treatments. Four replicates were assessed during this trial.

For each treatment, 30 million sperm cells were stained with Hoechst 33342 (H33342) in a total volume of 1200 µL of pNCM and subjected to bulk sorting. A total of 5,184,000 cells (max per 4 tube sort) were sorted per treatment and time point. The in vitro capacitation (IVC) group was incubated for 4 h and then assessed for the ability to capacitate. Recovery rates were quantified using a hemocytometer, where the number of cells recovered was calculated as a percentage of the number of cells reported as sorted. Sperm motility was assessed using CASA at 0 h and again after 4 h of IVC. Sperm capacitation status was determined at both timepoints using IBFC.

This experimental setup allowed for direct comparisons of recovery efficiencies, motility parameters, and capacitation status across treatments, providing insights into the impact of catch media composition on sperm functionality.

### 2.2 Semen Collection and Processing

Four commercial Duroc boars with known field fertility from the same private boar stud were used in this study. The boars were housed one boar per pen, collected once per 7‐day period, and fed a commercial boar diet to maintain weight. Semen doses in this study were in excess of industry production following established standard operating procedures and were not directly collected for this research. Boar semen was collected using the standard two‐gloved hand technique. Only ejaculates with > 80% motility at the time of collection were used. Sperm concentration was determined using a hemocytometer. The semen was immediately extended (e.g., < 5 min) a total of five times the volume of the semen with a long‐term extender, with the extender being within 2°C of the semen, and delivered to Iowa State University the same day. Upon arrival, the temperature of the samples was annotated, and extended semen was stored at 17°C. The semen was then analyzed within 24 h of collection for concentration and motility using CASA and adjusted for a final concentration of 30 million cells/mL for each boar.

### 2.3 CASA

For concentration and motility, a 1000 µL aliquot of each sample was placed in a 1.5 mL Eppendorf tube and warmed for 20 min at 37°C. Samples were then mixed thoroughly to ensure even distribution of cells, and then 3.5 µL of each aliquot was loaded onto a 20 µm disposable counting chamber from Minitube (Minitüb GmbH, Tiefenbach, Germany). Concentration and motility were then assessed using a Zeiss Axioscope 5 microscope (Carl Zeiss Microscopy, LLC, Oberkochen, Germany) fitted with a Basler Ace ac2440‐75uc camera (Basler AG, Ahrensburg, Germany) and a 10×/0.25 A‐Plan objective lens using MinitubeAndroVision software (Reference Module: 12,500/1000, Tiefenbach, Germany). The condenser with an aperture diaphragm was set at 1.

### 2.4 Media

*pNCM*: Non‐capacitation media containing NaCl, KCl, NaH2PO_4_, Na lactate, MgCl_2_‐6H_2_O, TL‐HEPES, Na‐pyruvate, sorbitol, gentamicin, penicillin G, PVA, and glucose were prepared in 1000 mL of deionized water (ddH_2_O), as previously described [20]. Vacuum filtration was performed into an appropriate container, and the pH of the solution was adjusted to 7.20 ± 0.02 and stored at 4°C until use. *Porcine capacitation media (pCM)*: Capacitation media were prepared on the same day of experiments by adding NaHCO_3_, pyruvic acid, CaCl_2,_ and BSA to 50 mL of the prepared pNCM, as previously described [20]. *Short‐term extender*: Beltsville Thawing Solution (BTS) short‐term extender, manufactured by Minitube, was used. The solution was prepared and handled according to the manufacturer's guidelines. *Long‐term extender*: AndroStar Premium long‐term extender, manufactured by Minitube, was used. The solution was prepared and handled according to the manufacturer's guidelines.

### 2.5 Capacitation Treatments

Spermatozoa were evaluated before (0 h, in pNCM) and after (4 h, in pCM) IVC. A total of 5,184,000 sperm cells were sorted per treatment group for immediate analysis (0 h) and for 4 h pCM IVC. Both 0 h and 4 h samples were initially assessed for motility using CASA. Following motility assessment, the 0 h samples were immediately prepared for IBFC analysis. For the 4 h samples, sperm cells were incubated in pCM at 37°C. After the 4 h incubation, motility was reassessed, and the samples were then prepared for IBFC.

### 2.6 Reagents

FluoZin‐3 (FZ3; zinc probe) from Invitrogen (2530139, Waltham, MA, USA) was reconstituted with DMSO to a stock solution of 500 µM. Lectin PNA (*A. hypogea*/peanut agglutinin) conjugated to Alexa Fluor 594 from Invitrogen (L32459) was reconstituted with ddH_2_O at a stock solution of 0.5 µg/mL. Hoechst 33342 (H33342) from Calbiochem (382065, San Diego, CA, USA) was reconstituted with ddH_2_O to a stock solution of 18 mM. Propidium iodide from Acros Organics (AC440300010, Geel, Belgium) was reconstituted with ddH_2_O to a stock solution of 1 mg mL^−1^. CellRox Orange (CRO; Oxidative stress probe) from Invitrogen (C10443) was reconstituted with DMSO to a stock solution of 2.5 mM. MitoTracker Deep Red from Invitrogen (M22426) was reconstituted in DMSO to a stock solution of 1 mM.

### 2.7 Flow Cytometry Probe Staining and Incubation

For FACS, sperm concentration was taken from CASA measurements to adjust 30 million cells in 600 µL of pNCM and stained with H33342 at a 1:1000 concentration from the above stock, incubated at room temperature in the dark. The samples were then centrifuged at 500 × *g* for 5 min, the supernatant removed, and spermatozoa were resuspended in 1200 µL of pNCM before sorting.

For IBFC, a total of 5,184,000 cells per sample were centrifuged at 500 × *g* for 5 min and resuspended with 100 µL of pNCM containing the following concentration of fluorescent probes diluted from the above stocks: H33342 (1:1000), propidium iodide (1:1000), FZ3 (1:500), and PNA‐AF594 (1:2000), CRO (1:1000), MitoTracker Deep Red (1:2000). Cells were then incubated for 30 min at room temperature in the dark. The samples had their probes removed by centrifuging, the supernatant removed and resuspended with 100 µL of phosphate‐buffered saline (PBS) without NaN_3_ for compatibility with IBFC. The samples were then incubated at 37°C for 30 min in the dark to allow complete de‐esterification of FZ3 intracellular acetoxymethyl (AM) esters. Finally, samples were run on the image‐based flow cytometer.

### 2.8 Fluorescence Activated Cell Sorting

Cell sorting was performed using the BD FACSMelody Cell Sorter (BD Life Sciences, San Jose, CA, USA). The instrument was equipped with three spatially separated excitation lasers: 405 nm violet laser (40 mW), 488 nm blue laser (20 mW), and 640 nm red laser (40 mW). The fluidics system utilized a quartz cuvette flow cell with gel‐coupled optics for optimal light collection and resolution. A 100 µm nozzle was used to ensure gentle handling of sperm cells. Flow speed was maintained at a setting of 10. Sample input was controlled via software‐adjustable agitation at 200 rpm to keep cells suspended. Sorted populations were collected in 5.0 mL polystyrene tubes. The photomultiplier tube (PMT) settings for the H33342 gated population was set at 55. The sorting process was managed using BD FACSChorus Software (v1.4.3.0), which provided automated setup and optimization of droplet breakoff, stream alignment, and sort delay using BD FACS Accudrop beads. Instrument performance was verified daily using BD CS&T quality control beads to ensure consistent fluorescence sensitivity and alignment. Forward scatter (FSC), side scatter (SSC), and the 405 nm violet laser were used to resolve sperm populations stained with H33342. Sort efficiency was between 85% and 95% for all sorts. Acquisition rates ranged between 8000 and 10,000 events per second. The sort rate ranged from 2000 to 3000 events per second. Post‐sort, samples were taken off the sorter and placed in a centrifuge at 500 × *g* for 20 min, supernatant removed, resuspended in 100 µL of their respective catch media, and placed in a 1.5 mL microcentrifuge tube in a 37°C heating block for 20 min before recovery and motility assessment. Samples sat no longer than 30 min in catch media while in the sorter for optimized throughput. See Figure S1 for gating structure.

### 2.9 Cell Recovery Assessment Using Hemocytometer

Sperm recovery rates were determined using a metallized Bright‐Line Hemocytometer (Hausser Scientific, Horsham, PA). Following FACS and centrifugation to concentrate the sperm cells, a 10 µL aliquot of the sorted sperm suspension was loaded into the hemocytometer chamber. The ruled surface of the hemocytometer, with a depth of 0.1 mm and a volume of 0.1 µL per square, was used to count sperm cells using a Leica DM500 microscope (Leica Microsystems, Wetzlar, Germany) equipped with a 20×/0.40 objective lens. Sperm counts were recorded, and recovery was calculated as the percentage of the total reported number of sperm sorted. Each sample was counted in duplicate to ensure accuracy, and the mean value was used for statistical analysis.

### 2.10 IBFC Data Acquisition

A Cytek Amnis ImageStreamX MkII Image‐Based flow cytometer (ISX, Fremont, CA, USA) fitted with a 40× microscope objective and an imaging rate up to 2000 events/s was used for phenotyping sperm biomarker defined status. The sheath fluid was phosphate‐buffered solution void of Mg^2+^ or Ca^2+^ with a pH of 7.20. The flow‐core diameter and speed were 6 µm and 66 mm/s, respectively. Raw image data were acquired using INSPIRE software version 3.0. In INSPIRE data acquisition software, the following channels were collected: two bright‐field channels were collected (Channels 1 and 9), an FluoZin 3 image (Channel 2), one CRO image (Channel 3), one Propidium Iodide image (Channel 5), one SSC image (Channel 6), one Hoechst 33342 image (Channel 7), one PNA‐AF594 image (Channel 10), and one MitoTracker Deep Red image (Channel 11). The following lasers and power settings were used: 405 nm (to excite H33342): 10 mW; 488 nm (to excite FluoZin 3 AM): 60 mW; 561 nm (to excite Propidium Iodide and CRO): 100 mW; 592 nm (to excite PNA‐AF594): 150 mW; 642 nm (to excite MitoTracker Deep Red): 150 mW; and 785 nm (SSC laser): 5 mW. SpeedBeads were used to ensure focus of the cells throughout acquisition.

### 2.11 IBFC Data Analysis

Data was analyzed using IDEAS analysis software version 6.4. The gating approach used standard focus and single cell gating calculations created by IDEAS software. Image‐based calculations were also used to discard spermatozoa laterally aligned with the camera, as opposed to anteriorly/posteriorly aligned, as previously described [37, 38].

### 2.12 Statistical Analysis

Statistical analyses were conducted in R (v4.2.2) using the nlme (v.3.1‐168) [39] and emmeans (v.1.11.0) [40] packages to evaluate the effects of treatment and timepoint on the measured parameters. A linear mixed‐effects model was fitted. In this model, *Treatment*, *Time*, and their interaction were included as fixed effects to estimate population‐level differences across conditions, while *Boar* was included as a random effect to account for repeated measures and variability attributable to individual animals. Heterogeneous variances across treatment groups were accommodated using a variance structure in the model. Model assumptions were assessed for each fitted model. All data points were retained for analysis to reflect the full biological variability of the dataset; no outliers were excluded. Post hoc pairwise comparisons between groups were performed using the emmeans package to compute estimated marginal means (least‐squares means). Tukey's Honestly Significant Difference (HSD) method was applied to adjust for multiple comparisons. Group differences were visualized using bar charts using the ggplot2 (v.3.5.1) [41] package with error bars representing the standard error of the mean (SEM). Statistical significance was assessed at *p *< 0.05, and results are reported with corresponding estimated marginal means and 95% confidence intervals.

## 3 Results

**FIGURE 1:**
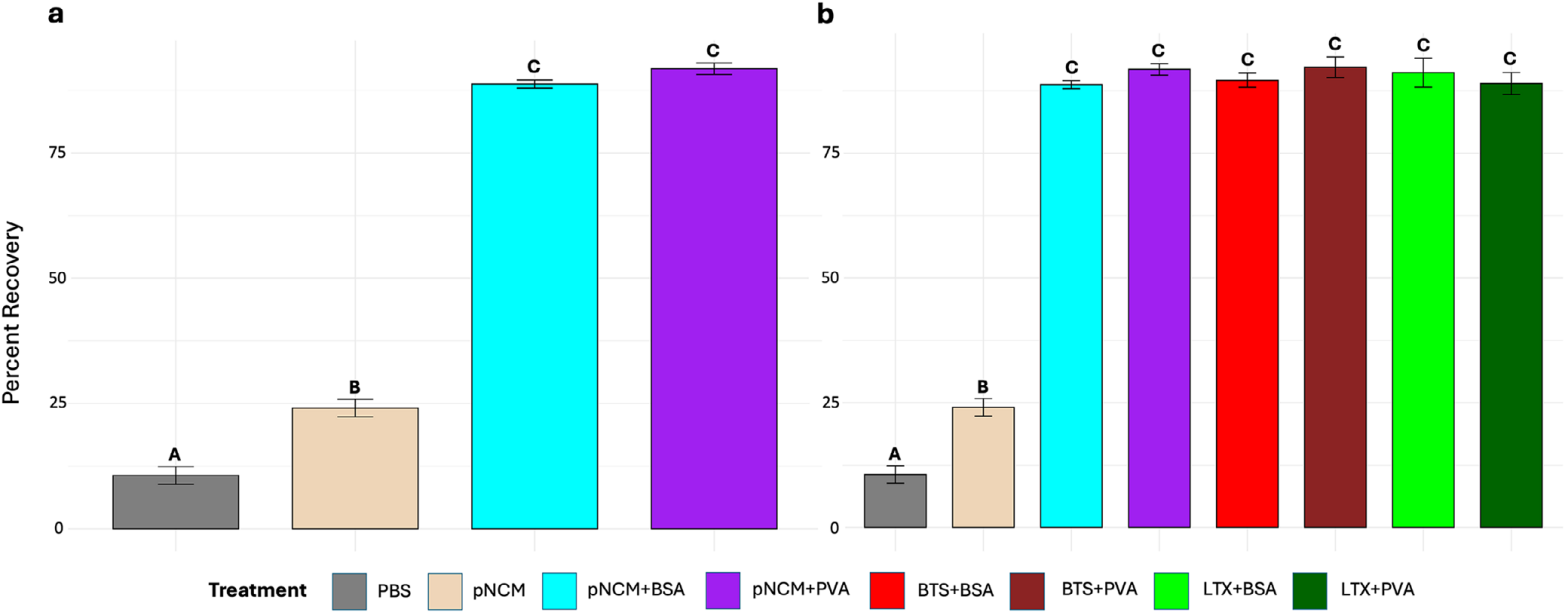
Quantification of sperm cell recovery across different catch media following flow cytometric sorting. Bar graphs display mean percent recovery ± SEM of spermatozoa post‐sorting under various catch media conditions, with treatments grouped by (a) a subset of PBS and pNCM with and without supplementation of BSA or PVA, and (b) a comprehensive panel of all eight treatments tested. Sperm recovery is defined as the percentage of sorted spermatozoa that were successfully collected and retained measurable motility after flow cytometric sorting relative to the total number of spermatozoa sorted. Letters above bars indicate statistically significant differences between treatments (*p *< 0.05, Tukey‐adjusted post hoc test).

Sperm recovery rates were quantified across all treatments and replicates using hemocytometer counts shown in Figure 1. Recovery was calculated as a percentage of the sperm cells sorted by treatment and timepoint. The sorted control group (PBS and pNCM without supplementation) showed negligible recovery (10.6% and 24.1% respectively). Among the sorted groups, all treatments with supplemented BTS or PVA catch media demonstrated high recovery rates, ranging from 88.8% to 92.2%. In treatments using pNCM as the catch media, supplementation with 1% BSA yielded an average recovery rate of 88.8%, and with 0.1% PVA resulted in a recovery of 91.8% (*p *> 0.05). BTS treatments showed comparable recovery rates, with supplementation with 1% BSA achieving 89.6% recovery and supplementation with 0.1% PVA achieving 92.2%. Similarly, treatments with LTX as the catch media had recovery rates of 91.1% recovery with 1% BSA, and 89.0% with 0.1% PVA.

**FIGURE 2:**
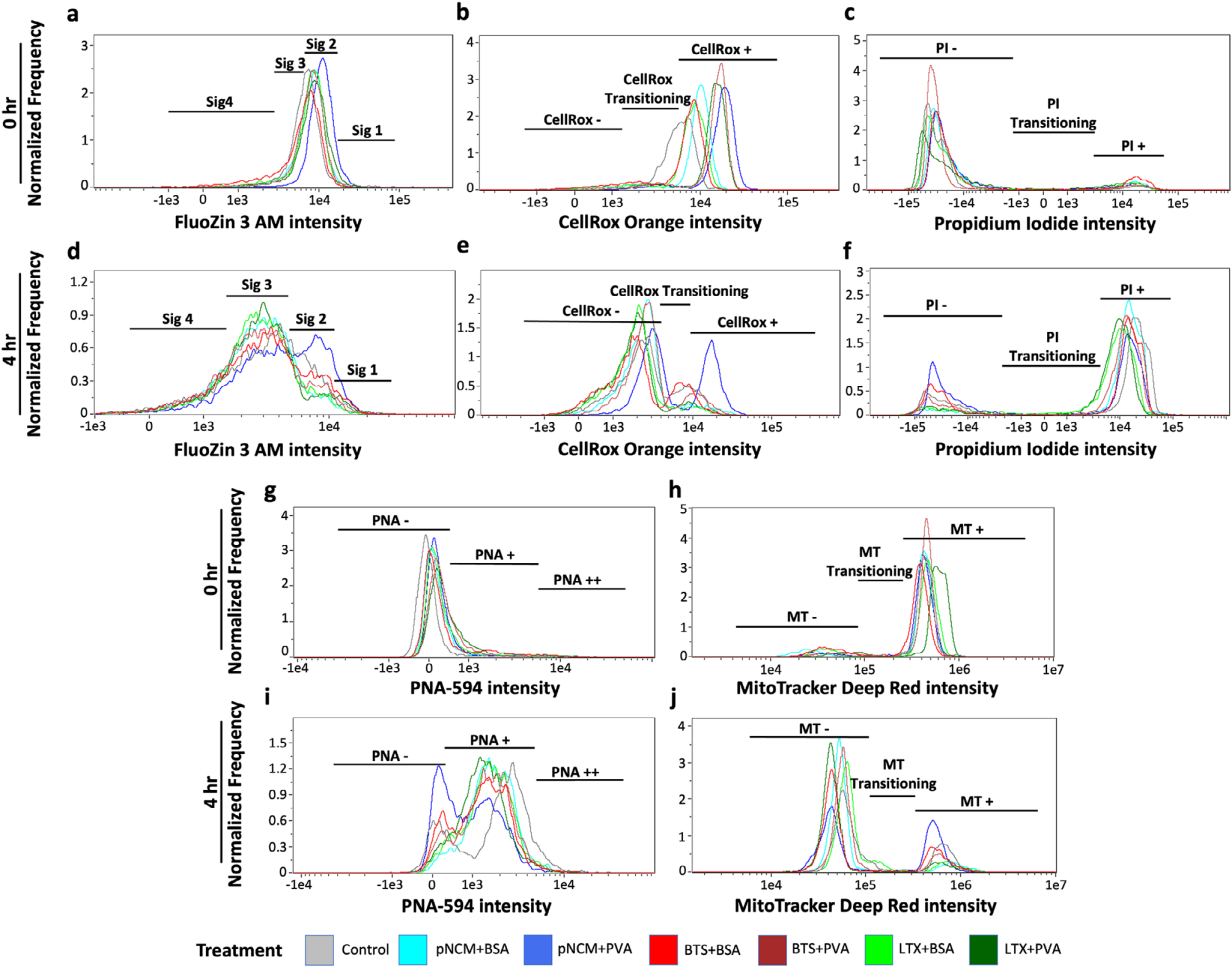
Overlay of Biomarker Fluorescence Intensity Distributions Across All Treatment Groups at 0 h and 4 h Timepoints. Image‐based flow cytometry (IBFC) was used to quantify the fluorescence intensities of spermatozoa stained with six fluorescent probes across seven treatment groups, including an unsorted control and six post‐sort media conditions (pNCM + BSA, pNCM + PVA, BTS + BSA, BTS + PVA, LTX + BSA, and LTX + PVA). Panels (a–c, g, h) show normalized fluorescence intensity distributions at 0 h, and panels (d–f, i, j) show corresponding distributions at 4 h. Boar #4 is displayed here for illustrative purposes. Biomarkers measured include: (a, d) FluoZin‐3 AM for zinc ion localization and zinc signature classification (Signatures 1–4); (b, e) CellRox Orange for reactive oxygen species detection (CellRox negative, transitioning, and CellRox Positive); (c, f) Propidium iodide (PI) for plasma membrane integrity (PI negative, transitioning, and PI positive); (g, i) PNA‐AF594 for acrosomal remodeling (PNA negative, PNA positive, and PNA positive‐positive); and (h, j) MitoTracker Deep Red for mitochondrial activity (MT negative, transitioning, and MT positive). Each histogram represents a normalized frequency distribution of fluorescent intensity for sperm populations within each treatment group at the indicated timepoint.

IBFC was used to compare the distribution of fluorescent biomarker intensities across seven treatment groups at two timepoints: 0 h and 4 h. This included an unsorted control and six post‐sort sperm samples recovered in different catch media: pNCM, BTS, or LTX supplemented with 1% BSA or 0.1% PVA. Fluorescence intensity distributions were overlaid for each biomarker to visualize differences across treatment groups and timepoints (Figure 2). Biomarkers included FluoZin‐3 AM (zinc ion localization, classified into four zinc signatures), CRO (ROS), Propidium iodide (plasma membrane integrity), PNA‐AF594 (acrosomal remodeling), and MitoTracker Deep Red (mitochondrial activity). Histograms for each marker showed a range of intensity profiles between treatments, with notable shifts in zinc signature classifications, ROS levels, membrane and acrosome integrity, and mitochondrial staining between 0 h and 4 h.

Motility parameters provide key insights into sperm functionality and are critical indicators of sperm quality during capacitation and fertilization processes. In this study, six general motility parameters were evaluated using CASA: total motility, progressive motility, rapid motility, progressive circular motility, slow motility, and local motility.

**FIGURE 3:**
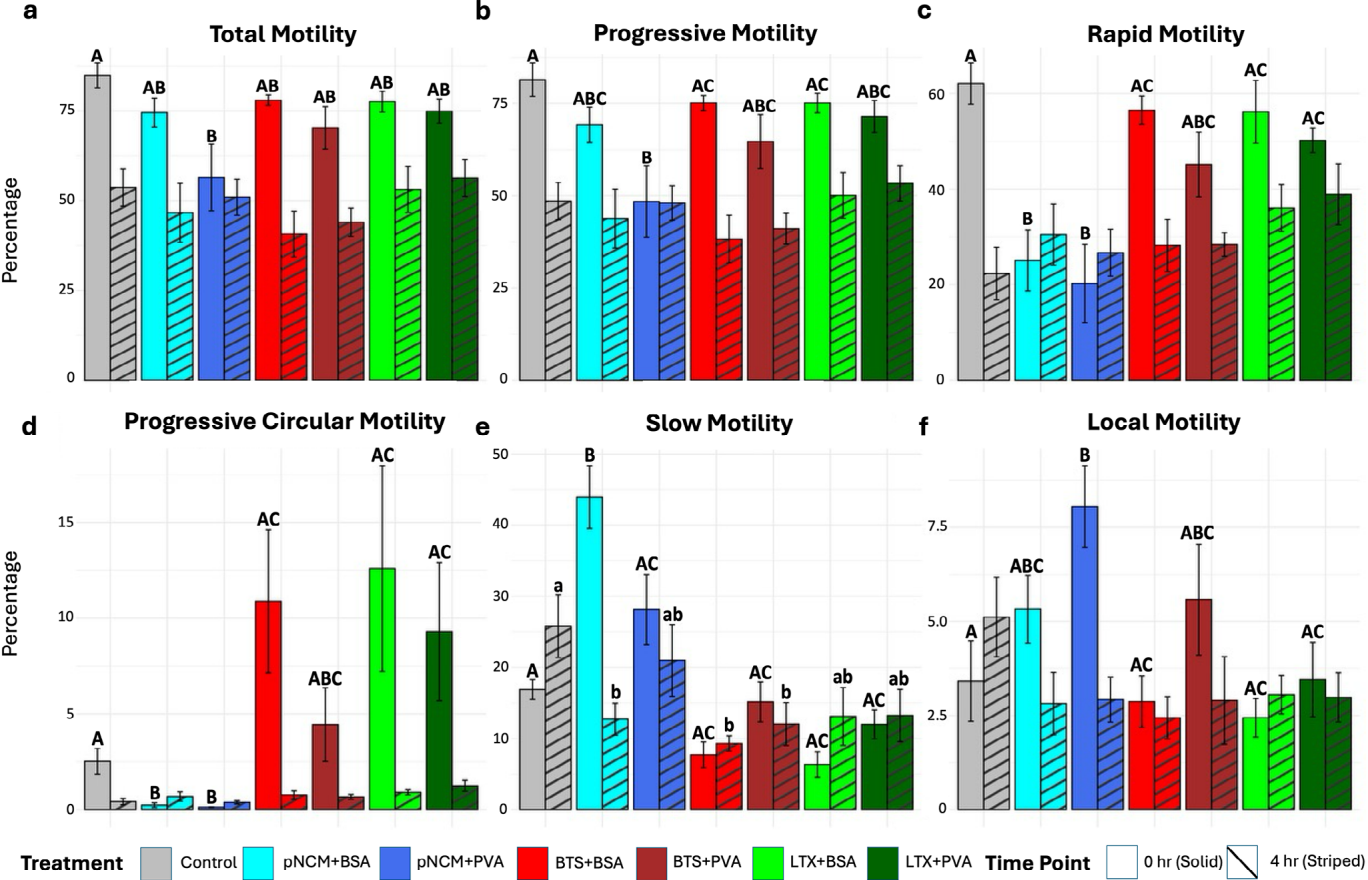
Motility parameters of spermatozoa across treatment groups after 4 h of in vitro capacitation. Bar plots display the mean ± SEM for six general motility parameters measured at 0 h (solid bars) and 4 h (striped bars) by CASA across seven treatment conditions: unsorted control and pNCM, BTS, and LTX supplemented with 1% BSA or 0.1% PVA. Motility parameters include: (a) total motility, (b) progressive motility, (c) rapid motility, (d) progressive circular motility, (e) slow motility, and (f) local motility. Letters above bars indicate statistically significant differences between treatments (*p *< 0.05, Tukey‐adjusted post hoc test), evaluated separately for each timepoint. Capital letters denote significance among treatments at 0 hr, and lowercase letters denote significance among treatments at 4 h. Bars without letters indicate no significant differences. See Figure S2 for differences across timepoints within treatment.

At the 0 h timepoint, total, progressive, and rapid motility were highest in the unsorted control group, which served as a baseline for comparison (Figure 3). Among the sorted treatments, pNCM supplemented with 0.1% PVA displayed lower averages compared to its 1% BSA counterpart and the unsorted control (*p *< 0.05), and both LTX treatments consistently outperformed the other sorted groups across these parameters. Progressive circular motility was low across both pNCM treatments, and higher values were observed in the BTS + 1% BSA, LTX + 1% BSA, and LTX + 0.1% PVA treatments. Slow motility was elevated in pNCM treatments compared to BTS and LTX treatments. Local motility was greatest in treatments supplemented with 0.1% PVA, with pNCM + 0.1% PVA having the highest amount present amongst all sorted treatments.

At the 4 h timepoint, total and rapid motility declined across all treatments compared to the 0 h measurements, which is normal after 4 h of IVC. All sorted treatments exhibited higher rapid motility than the unsorted control, and both pNCM groups had higher averages than 0 h treatments for this parameter. Progressive circular motility remained low across treatments, with a slight increase observed in LTX + 0.1% PVA. All sorted treatments had lower averages for slow motility compared to the unsorted control, with pNCM + 0.1% PVA having the highest average among the sorted treatments. Local motility decreased across all groups at 4 hr, with all sorted treatments showing lower values than the unsorted control.

**FIGURE 4:**
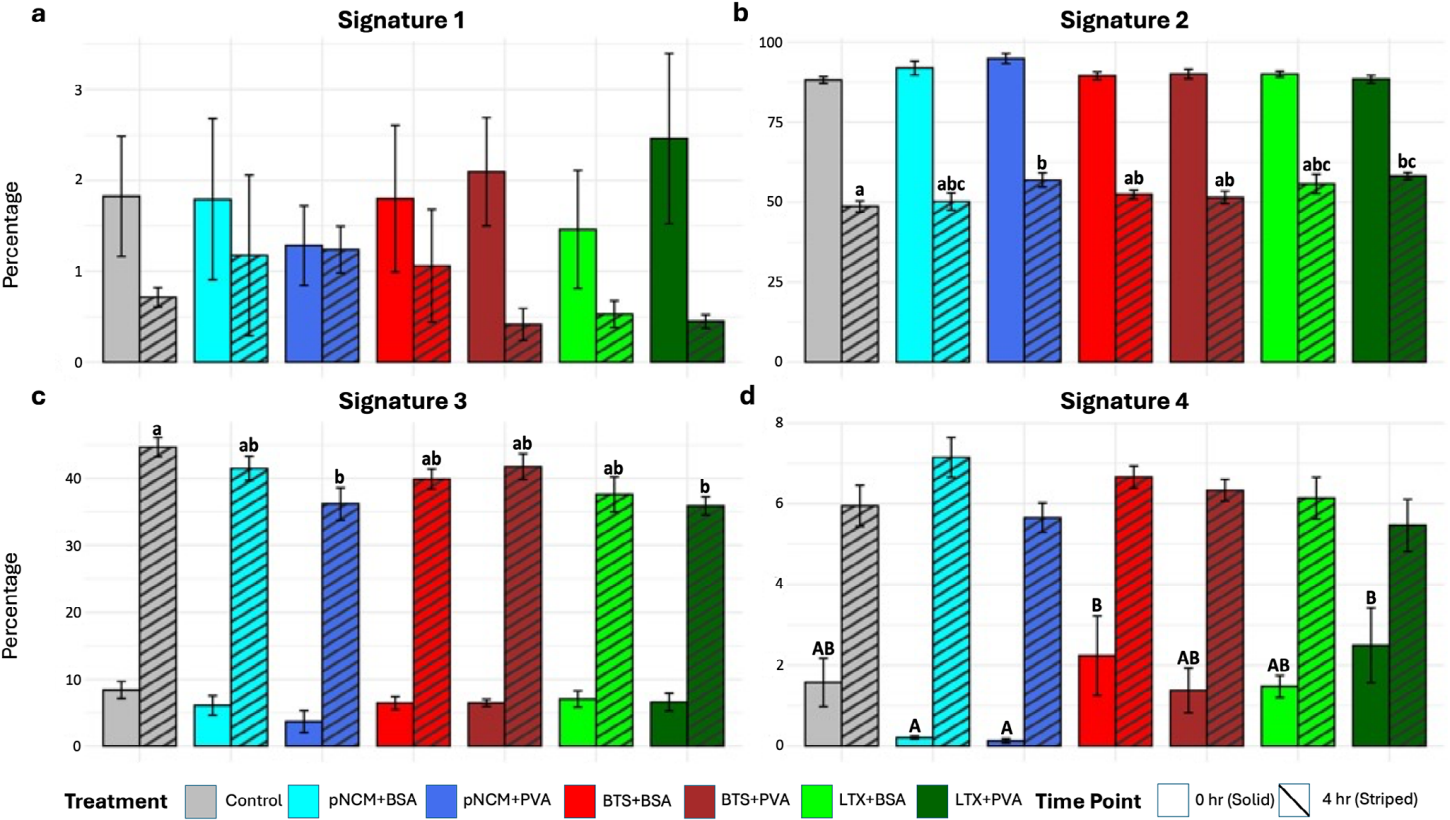
Distribution of zinc signatures in spermatozoa across treatments and timepoints as measured by image‐based flow cytometry. Bar plots display the mean ± SEM for four zinc signature populations at 0 h (solid bars) and 4 h (striped bars) across seven treatment groups: unsorted control and pNCM, BTS, and LTX supplemented with 1% BSA or 0.1% PVA. Zinc signatures, determined by FluoZin‐3 AM staining and fluorescence localization, include: (a) Signature 1, (b) Signature 2, (c) Signature 3, and (d) Signature 4. Letters above bars indicate statistically significant differences between treatments (*p *< 0.05, Tukey‐adjusted post hoc test), assessed separately for each timepoint. Capital letters denote significance among treatments at 0 h, and lowercase letters denote significance among treatments at 4 h. Bars without letters indicate no significant differences. See Figure S3 for differences across timepoints within treatment.

Zinc signature classification provided further resolution into capacitation‐associated membrane changes across treatment groups and timepoints (Figure 4). At the 0 h timepoint, zinc Signature 1 accounted for less than 3% of the sperm population and showed no significant differences between the non‐sorted control and the treatment groups. Zinc Signature 2 remained consistent across all treatment groups, representing 85–95% of the sperm population. Zinc Signature 3 exhibited little variation between the non‐sorted control and the treatment groups, while Signature 4 displayed a decrease in pNCM treatments and an increase in BTS + 1% BSA and LTX + 0.1% PVA treatments (*p *< 0.05).

At the 4 h timepoint, zinc Signature 1 continued to show no differences between the non‐sorted control and the treatment groups, although both pNCM treatments exhibited slightly elevated averages. Zinc Signature 2 demonstrated higher values for pNCM + 0.1% PVA and LTX + 0.1% PVA treatments compared to the non‐sorted control (*p *< 0.05). Zinc Signature 3 was significantly lower in pNCM + 0.1% PVA and LTX + 0.1% PVA compared to the non‐sorted control (*p* < 0.05). Other treatments showed numerically lower averages but did not differ statistically from control. Zinc Signature 4 showed no differences between the non‐sorted control and the treatment groups.

CRO, a fluorogenic probe used to detect ROS in sperm cells was used to evaluate oxidative status (Figure S4). At the 0 h timepoint, CellRox negative, transitioning, and positive populations showed no statistical differences between the non‐sorted control and the sorted treatments. After 4 h of IVC, the CellRox negative populations displayed higher non‐significant trends across all sorted treatments compared to the non‐sorted control, with LTX + 1% BSA showing the highest average among the sorted treatments (*p *> 0.05). CellRox transitioning populations remained relatively stable across treatments at both timepoints. CellRox positive populations were lower in all sorted treatments at 4 h compared to control, with LTX + 1% BSA showing the greatest numerical reduction (*p *> 0.05). However, no statistically significant differences were detected among any treatment groups for any CellRox population at either timepoint. See Figure S5 for differences across timepoints within treatment.

**FIGURE 5:**
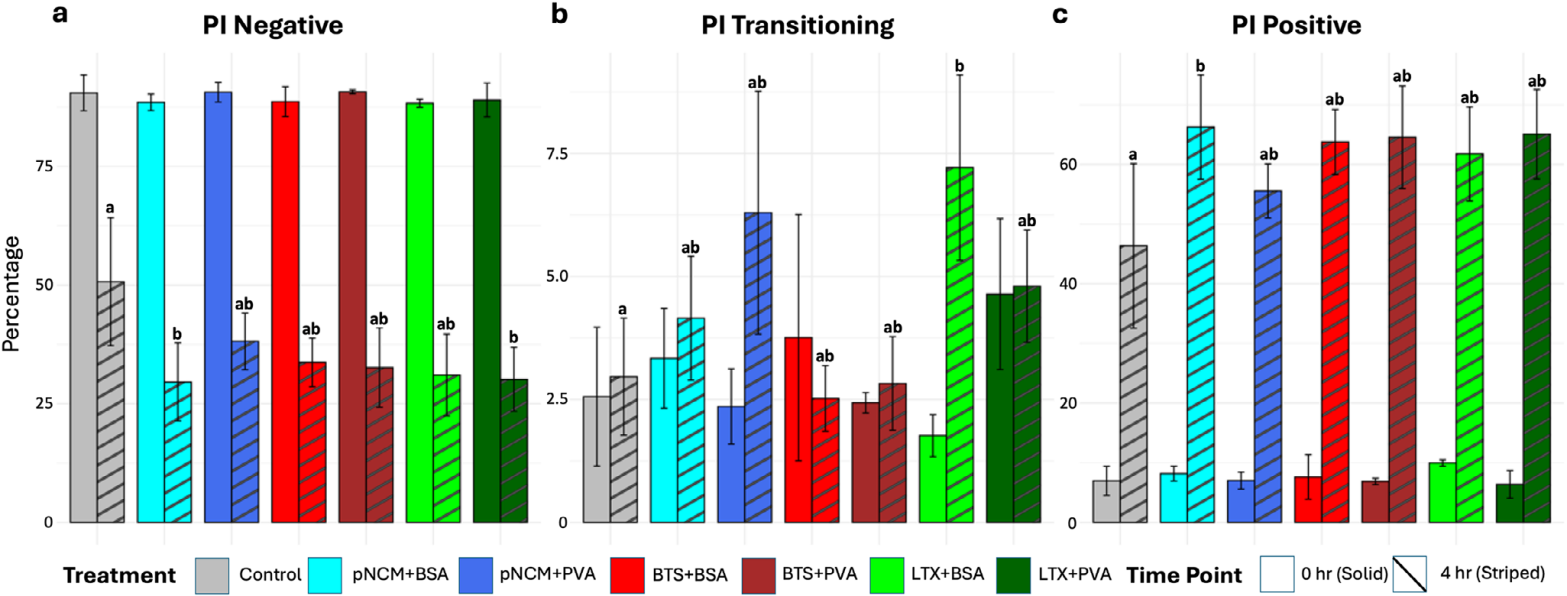
Assessment of plasma membrane integrity/remodeling in spermatozoa using propidium iodide (pi) fluorescence across treatments and timepoints. Bar plots display the mean ± SEM of sperm populations categorized by PI fluorescence intensity at 0 h (solid bars) and 4 h (striped bars) across seven treatment groups: unsorted control and pNCM, BTS, and LTX supplemented with 1% BSA or 0.1% PVA. PI fluorescence was used to evaluate plasma membrane integrity and/or remodeling, with spermatozoa classified as (a) PI Negative (intact/non‐remodeled membrane), (b) PI Transitioning (intermediate membrane permeability/remodeling), or (c) PI Positive (compromised/remodeled membrane). Statistically significant differences between treatments at the 4 h timepoint are indicated by lowercase letters above bars (*p *< 0.05, Tukey‐adjusted post hoc test). Bars sharing the same letter are not significantly different, while those with different letters differ significantly. Bars without letters indicate no significant differences. See Figure S6 for differences across timepoints within treatment.

Propidium iodide is a fluorescent dye that selectively binds to nucleic acids in cells with compromised or remodeled plasma membranes, serving as a marker of cell viability (Figure 5). At the 0 h timepoint there were no significant differences observed across the PI‐negative, PI‐transitioning, and PI‐positive populations among the non‐sorted control and treatment groups.

After 4 h of IVC, the PI‐negative populations numerically showed lower averages in all sorted treatment groups, with statistically significant reduction in pNCM + 1% BSA and LTX + 0.1% PVA treatments (*p *< 0.05). The PI‐transitioning populations exhibited elevated averages pNCM + 0.1% PVA and LTX + 1% BSA treatments relative to the non‐sorted control (*p *< 0.05). Additionally, all sorted treatments displayed numerically increased PI‐positive population averages, with pNCM + 1% BSA being significantly increased from the non‐sorted control (*p *< 0.05).

PNA‐AF594 (peanut agglutinin conjugated with Alexa Fluor 594) is a fluorescent probe that binds specifically to glycoconjugates containing β‐galactose residues, which are exposed on the outer acrosomal membrane of spermatozoa during capacitation and acrosomal exocytosis [42]. Across all timepoints, no significant differences were observed among the negative, positive, and positive‐positive PNA populations as shown in Figure S7. Minimal variation was found between PNA‐negative populations at the 0 h timepoint. At the 4 h timepoint, all sorted treatments displayed numerically lower PNA‐negative averages than the non‐sorted control but were not significantly different (*p *> 0.05). PNA‐positive populations exhibited elevated averages across all sorted treatments compared to the non‐sorted control, but were not significantly different (*p *> 0.05). See Figure S8 for differences across timepoints within treatment.

**FIGURE 6:**
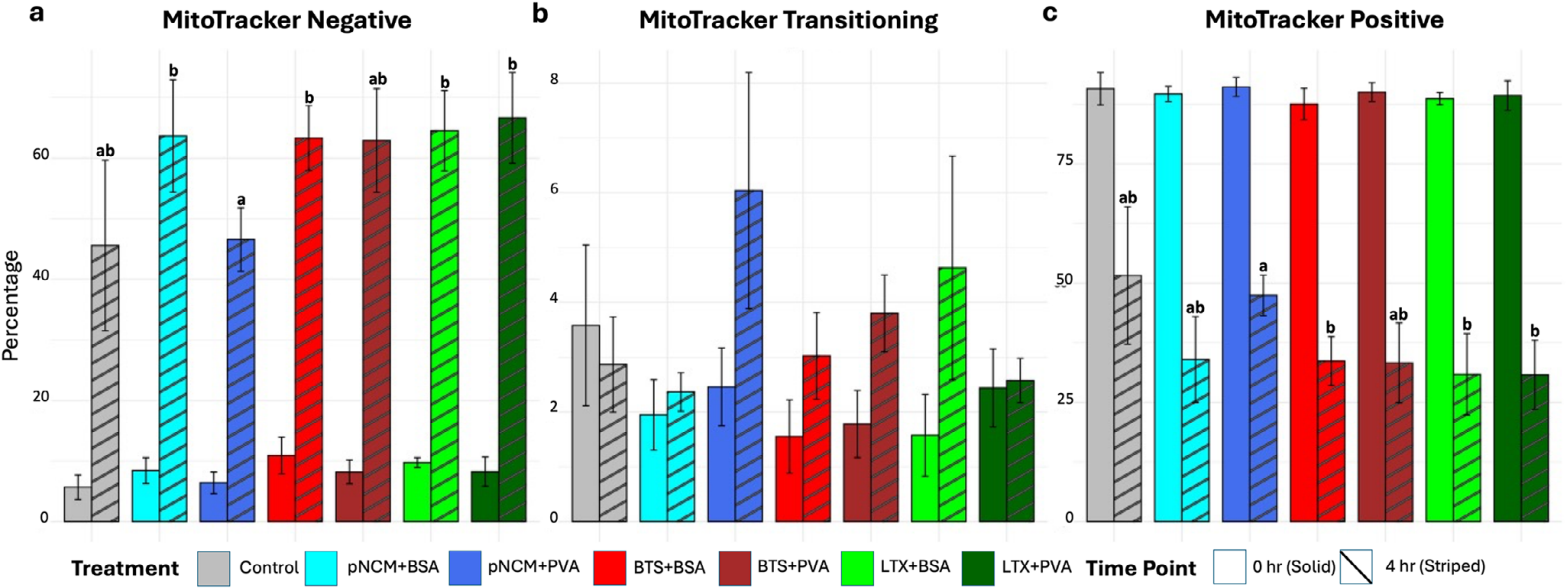
Analysis of mitochondrial activity in spermatozoa using MitoTracker Deep Red Fluorescence across treatments and timepoints. Bar plots display the mean ± SEM of sperm populations categorized by mitochondrial membrane potential at 0 h (solid bars) and 4 h (striped bars) across seven treatment groups: unsorted control and pNCM, BTS, and LTX supplemented with 1% BSA or 0.1% PVA. MitoTracker Deep Red fluorescence was used to evaluate mitochondrial activity, with spermatozoa classified as (a) MitoTracker Negative (low or absent mitochondrial activity), (b) MitoTracker transitioning (intermediate activity), and (c) MitoTracker positive (high mitochondrial activity). Statistically significant differences between treatments at the 4 h timepoint are indicated by lowercase letters above bars (*p *< 0.05, Tukey‐adjusted post hoc test). Bars sharing the same letter are not significantly different, while those with different letters differ significantly. Bars without letters indicate no significant differences. See Figure S9 for differences across timepoints within treatment.

MitoTracker Deep Red is a fluorescent probe used to evaluate mitochondrial activity and membrane potential in live cells. This dye selectively accumulates in active mitochondria with intact membrane potential, making it a reliable indicator of mitochondrial function and overall cell health. At the 0 h timepoint, no significant differences were observed among all MitoTracker negative, transitioning, and positive populations across all sorted treatments, as shown in Figure 6. MitoTracker Positive populations showed minimal variation from the non‐sorted control.

At the 4 h timepoint, MitoTracker negative populations exhibited elevated averages in all sorted treatments compared to the non‐sorted control except for pNCM + 0.1% PVA, which remained comparable. MitoTracker transitioning populations displayed no significant differences from sorted treatments to the non‐sorted control. MitoTracker positive populations decreased across all sorted treatments compared to the non‐sorted control, with both LTX treatment groups displaying the most significant differences.

## 4 Discussion

Advancements in fluorescence‐based technologies, such as IBFC and FACS, have revolutionized the assessment of cellular health and functionality as well as enabled sorting of cells. These tools offer high‐resolution analyses of critical biomarkers, enabling precise evaluation of physiological states such as capacitation, membrane permeability, acrosome integrity, and mitochondrial activity. Flow cytometry has been widely used to assess cellular populations for therapeutic applications, including sperm selection for ART [43, 44]. Cell sorting potential extends beyond clinical applications to agricultural settings, where optimizing sperm functionality and fertilization success is essential for improving livestock production originating from sorting [1, 3]. By integrating these technologies into agricultural workflows, researchers can enhance targeted breeding strategies, improve reproductive efficiency, and reduce resource waste associated with suboptimal fertilization.

High recovery rates across all supplemented catch media emphasize the importance of proper media composition for maintaining sperm viability post‐sorting. With each treatment starting from 5,184,000 sorted cells, consistent recoveries between 88.8% and 92.2% suggest that the sorting process had minimal impact on cell loss when appropriate media was used. In contrast, the low recovery in non‐PVA or BSA supplemented controls (PBS and pNCM alone) underscores the need for sufficient catch media support. Some cell loss in the supplemented catch media is likely result from handling steps (e.g., centrifugation).

Sperm motility is a fundamental pre‐requisite of fertilization success, directly influencing a sperm cell's ability to navigate the female reproductive tract and reach the oocyte [45]. This study quantified six general motility parameters, including total, progressive, rapid, progressive circular, slow, and local motility between each treatment and timepoint. These parameters quantify overall movement but also highlight specific motility patterns, offering insight into treatment effects on sperm quality and functionality. LTX and BTS treatments consistently preserved total, progressive, and rapid motility, with minimal deviation from the unsorted control. In contrast, pNCM treatments showed reduced performance, with lower motility and higher levels of slow and local movement, suggesting limited protective capacity during sorting and after 4 h of IVC. While progressive circular motility was generally low, significant increases in BTS + 1% BSA and both LTX treatments at 0 h may reflect early sorting responses. These findings underscore the importance of optimizing catch media to preserve sperm quality during and after sorting and the role of appropriately prepared media in preserving motility and sperm function [46].

Zinc efflux is a hallmark of sperm capacitation, marking the transition of spermatozoa from a stable, non‐capacitated state to a sperm cell prepared for fertilization. The removal of zinc destabilizes the sperm plasma membrane, increasing its fluidity and permeability, which are essential for downstream events such as hyperactivation and the acrosome reaction as previously described [20]. Zinc signatures reflect the dynamic changes in zinc ion distribution during sperm capacitation, a process critical for fertilization [21, 38]. In this study, FluoZin‐3 AM was used to assess zinc localization as a biomarker of capacitation status under various treatment conditions. This approach allowed us to delineate capacitation‐associated membrane changes in sorted spermatozoa or if the spermatozoa could still progress through sperm capacitation normally.

The most notable deviations between treatment groups in zinc signatures were observed after 4 h IVC, primarily in Signatures 2 and 3. We observed more spermatozoa in a Signature 2 state (non‐capacitated) for pNCM + 0.1% PVA as compared to control (*p *< 0.05) after 4 h IVC as well as less spermatozoa achieving a capacitated, Signature 3 state (*p *< 0.05), meaning less spermatozoa progressed in capacitation. Signature 2, indicative of less capacitated state, exhibited a non‐significant increase across all treatments except pNCM + 1% PVA, compared to the non‐sorted control, suggesting similar levels of capacitation occurred in sorted spermatozoa. Signature 3, associated with mid to late stages of sperm capacitation, displayed no difference at 0 h. Signature 3 (more capacitated) after 4 h of IVC had some treatments with significantly lower averages such as pNCM + 1% PVA and LTX + 1% PVA. This indicates that while there was no sorting‐induced capacitation initially, as it relates to zinc Signature 3, less spermatozoa were capable of achieving mid to late‐stage capacitation, as defined by zinc Signature 3, after sorting with 4 h IVC.

Differences were observed in signature 4, representing fully capacitated spermatozoa, within the 0 h non‐capacitated treatments. While most treatments remained consistent with the non‐sorted control, BTS + 1% BSA and LTX + 0.1% PVA treatments showed significant increases from the pNCM treatments. Altogether with the other signatures, these findings suggest that zinc efflux dynamics are influenced by sorting and media conditions and is novel given there are no known literature on how the zinc localization is affected by the sorting process.

Given the critical role of membrane destabilization during capacitation, we next examined whether oxidative stress might also be altered in sorted spermatozoa. ROS are tightly regulated during capacitation, serving both signaling and damaging roles depending on their concentration and timing. Therefore, in addition to evaluating zinc efflux dynamics, we assessed oxidative stress markers using CRO [47] to determine whether sorting procedures or catch media influenced redox status after 4 h IVC.

Oxidative stress plays a critical role in both physiological and pathological processes of spermatozoa across many different species, including human, bull, and boar [48, 49, 50, 51]. While controlled levels of ROS are essential for capacitation, including hyperactivation and acrosomal exocytosis, excessive ROS generation can lead to lipid peroxidation, DNA damage, and loss of sperm motility, ultimately compromising fertilization potential [52, 53]. By using CRO in this study, we aimed to evaluate the oxidative status of sorted sperm populations across different catch media treatments and incubation conditions.

The consistent levels of CRO negative populations at the 0 h timepoint across all treatments suggests that the sorting process itself does not induce immediate oxidative stress, which contrasts other reports of induced ROS production from cell sorting in stallion using CellRox probe [54]. This could be from the lower fluidic pressure of the BD FACSMelody (23 psi) compared to sorting flow cytometers (40–50 psi) used in prior studies [13, 55]. Transitioning populations remained consistently low across both timepoints, indicating minimal oxidative stress in intermediate states. While not significant, the slight numerical elevation in transitioning populations for LTX + 0.1% PVA at the 4 h timepoint may hint at subtle treatment‐specific responses to incubation conditions.

Capacitation is accompanied by controlled remodeling of the sperm plasma membrane, including changes in permeability and fluidity that precede acrosomal exocytosis. To evaluate whether sorting induces premature or aberrant membrane remodeling consistent with capacitation, propidium iodide staining was used. Although commonly employed to detect loss of viability (live/dead), PI can also serve as a marker of plasma membrane destabilization [56, 57, 58] which can occur with capacitation. Assessing PI uptake allowed us to determine whether FACS or media conditions triggered capacitation‐like plasma membrane remodeling or contributed to plasma membrane damage after 4 h of IVC.

While sorting itself had minimal impact on membrane integrity at 0 h, the subsequent incubation period at 4 h revealed a decline in viability/plasma membrane integrity for all sorted treatments across both negative and positive PI populations, with significant differences in treatments such as pNCM + 1% BSA and LTX + 0.1% PVA. This suggests that sorting may exacerbate stress over time leading to increased membrane damage or induce increased susceptibility to plasma membrane remodeling during IVC. The non‐significant increases in positive PI populations at 4 h for all sorted treatments highlight the possible susceptibility of spermatozoa to membrane destabilization during the sorting process and prolonged incubation. These findings suggest that while this sorting protocol is generally effective at maintaining initial sperm integrity immediately after sorting (0 h), after 4hr of IVC, the plasma membrane is altered in sorted treatments compared to the non‐sorted control.

Having evaluated plasma membrane remodeling and stability, we next assessed acrosomal integrity using PNA‐AF594 labeling. PNA specifically binds to exposed glycoproteins on the inner acrosomal membrane, which become accessible after the acrosomal membrane is disrupted during capacitation or acrosome reaction [37, 59, 60]. In this study, PNA‐AF594 was employed to evaluate acrosomal integrity and potential capacitation‐related changes in porcine spermatozoa across treatments and timepoints. By identifying populations with differing acrosomal populations, we aimed to determine how sorting, media composition, and IVC afterwards influence acrosomal status, a critical factor for successful fertilization.

The results suggest that the experimental conditions and sorting treatments had minimal impact on the acrosomal status of the sperm populations immediately after sorting (0 h). At the 4 h timepoint, no significant differences were observed between the non‐sorted control and the sorted treatment groups (*p *> 0.05). This is inconsistent with previous bull and stallion literature that sorted spermatozoa have a lower percentage of acrosomal integrity [34, 61]. We might see a significant difference with increased power.

Beyond membrane changes, sperm functionality also depends heavily on mitochondrial energy production. Therefore, mitochondrial membrane potential was assessed using MitoTracker Deep Red to evaluate whether sorting and media conditions influenced sperm bioenergetics. MitoTracker Deep Red has been used to assess mitochondrial activity in spermatozoa across varying species of agricultural livestock [62, 63, 64]. By identifying populations with low and high mitochondrial activity, we aimed to determine how sorting conditions and catch media composition influence sperm bioenergetics during capacitation.

The consistency in the MitoTracker negative populations at the 0 h timepoint suggests that sorting had minimal immediate impact on mitochondrial activity. However, the observed increase in averages of the 4 h MitoTracker negative populations across some of the sorted treatments suggests a potential shift in mitochondrial activity during IVC, which may reflect a decline in metabolic activity in the sorted groups. Positive populations of some treatments declined at 4 h in all sorted treatments, indicating reduced mitochondrial activity compared to the non‐sorted control. This decline could suggest that sorting, even with optimized catch media, impose metabolic stress on sperm cells potentially impairing their energy production after 4 h of IVC. These findings emphasize the need for further optimization of sorting protocols and media compositions to maintain mitochondrial functionality during extended processing times, which is critical for ensuring sperm viability and fertilization potential in both agricultural and clinical settings.

## 5 Conclusion

The findings of this study highlight the nuanced effects of FACS and catch media composition on sperm recovery, motility, and capacitation status. While LTX‐based media consistently preserved motility metrics and recovery rates, variations in capacitation markers such as zinc efflux, oxidative stress, and mitochondrial activity revealed subtle treatment‐dependent effects compared to non‐sorted controls. Notably, the stability of acrosomal integrity and membrane permeability across treatments suggests that the sorting process, paired with optimized catch media, can maintain essential sperm parameters immediately after sorting but may impact the capacitation status after 4 h IVC. However, the observed declines in mitochondrial activity and membrane integrity after in vitro capacitation underscore the need for continued refinement of sorting protocols and media formulations. At the 0 h timepoint, sorted spermatozoa exhibited minimal deviations from the non‐sorted control in both motility metrics and capacitation biomarkers. However, after 4 h of IVC post‐sort, subtle shifts in mitochondrial activity, plasma membrane integrity, and zinc efflux suggest that sorting can influence capacitation dynamics in a treatment‐dependent manner. These insights contribute to the growing body of knowledge on sperm preservation technologies, with implications for improving reproductive efficiency in both agricultural and clinical applications. Future research should explore additional media formulations and conditions to mitigate the metabolic and physiological stressors, further enhancing the utility of FACS in targeted breeding strategies and assisted reproductive technologies.

## Author Contributions

*Conceptualization*: Tyler Weide and Karl Kerns. *Methodology*: Tyler Weide, Juan Steibel, and Karl Kerns. *Validation*: Tyler Weide, Juan Steibel, and Karl Kerns. *Data curation*: Tyler Weide. *Writing*: Tyler Weide, Juan Steibel, and Karl Kerns. *Formal analysis*: Tyler Weide, Juan Steibel, and Karl Kerns. *Review and editing*: Tyler Weide, Juan Steibel, and Karl Kerns. *Visualization*: Tyler Weide and Juan Steibel. *Supervision*: Juan Steibel, Karl Kerns. *Project administration*: Tyler Weide, Juan Steibel, and Karl Kerns. *Funding acquisition*: Karl Kerns. All authors have read and agreed to the published version of the manuscript.

## Ethics Statement

Ethical review and approval were not applicable for this study. This study involved the use of boar sperm samples, which were obtained from industry partners. The samples were not collected specifically for scientific research but were excess materials from standard production processes. The collection and handling of these samples adhered to standard industry protocols, designed to ensure ethical and humane treatment of the animals. Given the nature of the semen sample collection (a byproduct of routine production activities) and its alignment with standard agricultural practices, this study is exempt from Iowa State University Institutional Animal Care and Use Committee (IACUC) oversight.

## Conflicts of Interest

The authors declare no conflicts of interest.

## Supporting information




**Supplementary Figure 1**: Gating Strategy Used For Flow Cytometric Analysis of Sperm Populations. (a) Initial gating of all recorded events based on forward scatter area (FSC‐A) versus side scatter area (SSC‐A) to define the general sperm population *Scatter* and exclude debris and noise. (b) Gating on SSC‐H versus SSC‐W was performed to isolate singlets from doublets and aggregates based on side scatter properties *SSC Singlets*. (c) From the SSC singlet population, further refinement was done using FSC‐H versus FSC‐W to identify sperm singlets based on forward scatter pulse width and height *FSC Singlets*. (d) Final gating based on Hoechst 33342 fluorescence (H33342‐A) versus FSC‐H was used to select sperm populations that were successfully stained with DNA‐binding dye, further ensuring singlet identity and cell integrity. Events outside the DNA‐positive gate were excluded from downstream analysis.
**Supplementary Figure 2**: Change in Motility Parameters of Spermatozoa Across Treatment Groups Following 4‐Hour IVC Incubation. Bar plots display the mean ± SEM for six general motility parameters, representing the percent change from 0 hr to 4 hr (Δ 4hr – 0hr) following in vitro incubation in seven treatment conditions: unsorted control, pNCM, BTS, and LTX media, with each sorted treatment supplemented with either 1% BSA or 0.1% PVA. Motility parameters include: (a) Total Motility, (b) Progressive Motility, (c) Rapid Motility, (d) Progressive Circular Motility, (e) Slow Motility, and (f) Local Motility, as assessed by CASA. Letters above bars indicate statistically significant differences between treatments (P<0.05, Tukey‐adjusted post hoc test). Bars sharing the same letter are not significantly different.
**Supplementary Figure 3**: Change in Zinc Signature Profiles of Spermatozoa Across Treatment Groups Following 4‐Hour IVC Incubation. Bar plots display the mean ± SEM for four zinc signature populations (Δ 4hr – 0hr, %) across seven treatment conditions: unsorted control, pNCM, BTS, and LTX media, with each sorted treatment supplemented with either 1% BSA or 0.1% PVA. Zinc signature populations were quantified using Image‐Based Flow Cytometry and include: (a) Signature 1, (b) Signature 2, (c) Signature 3, and (d) Signature 4. Letters above bars indicate statistically significant differences between treatments (P<0.05, Tukey‐adjusted post hoc test). Bars sharing the same letter are not significantly different. Bars without letters indicate no significant differences.
**Supplementary Figure 4**: Quantification of Reactive Oxygen Species (ROS) in Spermatozoa Using CellRox Orange Fluorescence Across Treatments and Timepoints. Bar plots display the mean ± SEM of sperm populations classified by oxidative status at 0 hr (solid bars) and 4 hr (striped bars) across seven treatment groups: unsorted control and pNCM, BTS, and LTX supplemented with 1% BSA or 0.1% PVA. Oxidative status was determined based on CellRox Orange fluorescence intensity and classified into three categories: (a) CellRox Negative, (b) CellRox Transitioning, and (c) CellRox Positive. Bars without letters indicate no significant differences.
**Supplementary Figure 5**: Change in Oxidative Stress Profiles of Spermatozoa Across Treatment Groups Following 4‐Hour IVC Incubation. Bar plots display the mean ± SEM for three CellRox Orange‐defined populations (Δ 4hr – 0hr, %) across seven treatment conditions: unsorted control, pNCM, BTS, and LTX media, with each sorted treatment supplemented with either 1% BSA or 0.1% PVA. Oxidative stress status was assessed by Image‐Based Flow Cytometry and categorized into: (a) CellRox Negative, (b) CellRox Transitioning, and (c) CellRox Positive populations. Bars represent the change in frequency of each population from 0 hr to 4 hr incubation. Bars without letters indicate no significant differences.
**Supplementary Figure 6**: Change in Plasma Membrane Integrity of Spermatozoa Across Treatment Groups Following 4‐Hour IVC Incubation. Bar plots display the mean ± SEM for three PI‐defined populations (Δ 4hr – 0hr, %) across seven treatment conditions: unsorted control, pNCM, BTS, and LTX media, with each sorted treatment supplemented with either 1% BSA or 0.1% PVA. Membrane integrity was assessed using propidium iodide (PI) fluorescence via Image‐Based Flow Cytometry, and populations were categorized as: (a) PI Negative (intact membrane), (b) PI Transitioning (partial compromise), and (c) PI Positive (membrane‐compromised). Bars represent the change in frequency of each population from 0 hr to 4 hr incubation. Bars without letters indicate no significant differences.
**Supplementary Figure 7**: Evaluation of Acrosomal Remodeling in Spermatozoa Using PNA‐AF594 Fluorescence Across Treatments and Timepoints. Bar plots display the mean ± SEM of sperm populations stained with PNA‐AF594 at 0 hr (solid bars) and 4 hr (striped bars) across seven treatment groups: unsorted and pNCM, BTS, and LTX supplemented with 1% BSA or 0.1% PVA. Lectin peanut agglutinin (PNA) conjugated to AlexaFluor 594 was used to assess acrosomal integrity, with sperm classified as (a) PNA Negative (intact acrosome), (b) PNA Positive (remodeled/compromised acrosome), and (c) PNA Positive–Positive (extensive or advanced acrosomal remodeling/compromised). Bars without letters indicate no significant differences.
**Supplementary Figure 8**: Change in Acrosomal Status of Spermatozoa Across Treatment Groups Following 4‐Hour IVC Incubation. Bar plots display the mean ± SEM for three PNA‐AF594‐defined populations (Δ 4hr – 0hr, %) across seven treatment conditions: unsorted control, pNCM, BTS, and LTX media, with each sorted treatment supplemented with either 1% BSA or 0.1% PVA. Acrosomal membrane status was assessed by Image‐Based Flow Cytometry using peanut agglutinin (PNA) conjugated to Alexa Fluor 594, with populations defined as: (a) PNA Negative (intact acrosome), (b) PNA Positive (partial acrosome reaction), and (c) PNA Positive‐Positive (complete acrosome reaction). Bars represent the change in frequency of each population from 0 hr to 4 hr incubation. Bars without letters indicate no significant differences.
**Supplementary Figure 9**: Change in Mitochondrial Membrane Potential of Spermatozoa Across Treatment Groups Following 4‐Hour IVC Incubation. Bar plots display the mean ± SEM for three MitoTracker Deep Red‐defined populations (Δ 4hr – 0hr, %) across seven treatment conditions: unsorted control, pNCM, BTS, and LTX media, with each sorted treatment supplemented with either 1% BSA or 0.1% PVA. Mitochondrial membrane potential was assessed using MitoTracker Deep Red via Image‐Based Flow Cytometry, with populations defined as: (a) MitoTracker Negative, (b) MitoTracker Transitioning, and (c) MitoTracker Positive. Bars represent the change in frequency of each population from 0 hr to 4 hr incubation. Bars without letters indicate no significant differences among treatments.
