# Supplementary material for: Impact of Sorting and Catch Media on Porcine Sperm Motility, Capacitation, and Viability
Source: Andrology. 2026 Jan 28;14(7):2207–20. doi: 10.1111/andr.70184 (PMC13592060; doi:10.1111/andr.70184)
Supplement: Supplementary file 1 [file ANDR-14-2207-s001.docx]

**Supplemental Information**

**Supplementary Figure 1**. **Gating Strategy Used For Flow Cytometric Analysis of Sperm Populations.** (a) Initial gating of all recorded events based on forward scatter area (FSC-A) versus side scatter area (SSC-A) to define the general sperm population *Scatter* and exclude debris and noise. (b) Gating on SSC-H versus SSC-W was performed to isolate singlets from doublets and aggregates based on side scatter properties *SSC Singlets*. (c) From the SSC singlet population, further refinement was done using FSC-H versus FSC-W to identify sperm singlets based on forward scatter pulse width and height *FSC Singlets*. (d) Final gating based on Hoechst 33342 fluorescence (H33342-A) versus FSC-H was used to select sperm populations that were successfully stained with DNA-binding dye, further ensuring singlet identity and cell integrity. Events outside the DNA-positive gate were excluded from downstream analysis.

**Supplementary Figure 2. Change in Motility Parameters of Spermatozoa Across Treatment Groups Following 4-Hour IVC Incubation.** Bar plots display the mean ± SEM for six general motility parameters, representing the percent change from 0 hr to 4 hr (Δ 4hr – 0hr) following in vitro incubation in seven treatment conditions: unsorted control, pNCM, BTS, and LTX media, with each sorted treatment supplemented with either 1% BSA or 0.1% PVA. Motility parameters include: (a) Total Motility, (b) Progressive Motility, (c) Rapid Motility, (d) Progressive Circular Motility, (e) Slow Motility, and (f) Local Motility, as assessed by CASA. Letters above bars indicate statistically significant differences between treatments (P<0.05, Tukey-adjusted post hoc test). Bars sharing the same letter are not significantly different.

**Supplementary Figure 3. Change in Zinc Signature Profiles of Spermatozoa Across Treatment Groups Following 4-Hour IVC Incubation.** Bar plots display the mean ± SEM for four zinc signature populations (Δ 4hr – 0hr, %) across seven treatment conditions: unsorted control, pNCM, BTS, and LTX media, with each sorted treatment supplemented with either 1% BSA or 0.1% PVA. Zinc signature populations were quantified using Image-Based Flow Cytometry and include: (a) Signature 1, (b) Signature 2, (c) Signature 3, and (d) Signature 4. Letters above bars indicate statistically significant differences between treatments (P<0.05, Tukey-adjusted post hoc test). Bars sharing the same letter are not significantly different. Bars without letters indicate no significant differences.

**Supplementary Figure 4. Quantification of Reactive Oxygen Species (ROS) in Spermatozoa Using CellRox Orange Fluorescence Across Treatments and Timepoints.** Bar plots display the mean ± SEM of sperm populations classified by oxidative status at 0 hr (solid bars) and 4 hr (striped bars) across seven treatment groups: unsorted control and pNCM, BTS, and LTX supplemented with 1% BSA or 0.1% PVA. Oxidative status was determined based on CellRox Orange fluorescence intensity and classified into three categories: (a) CellRox Negative, (b) CellRox Transitioning, and (c) CellRox Positive. Bars without letters indicate no significant differences.

**Supplementary Figure 5. Change in Oxidative Stress Profiles of Spermatozoa Across Treatment Groups Following 4-Hour IVC Incubation.**
Bar plots display the mean ± SEM for three CellRox Orange-defined populations (Δ 4hr – 0hr, %) across seven treatment conditions: unsorted control, pNCM, BTS, and LTX media, with each sorted treatment supplemented with either 1% BSA or 0.1% PVA. Oxidative stress status was assessed by Image-Based Flow Cytometry and categorized into: (a) CellRox Negative, (b) CellRox Transitioning, and (c) CellRox Positive populations. Bars represent the change in frequency of each population from 0 hr to 4 hr incubation. Bars without letters indicate no significant differences.

**Supplementary Figure 6. Change in Plasma Membrane Integrity of Spermatozoa Across Treatment Groups Following 4-Hour IVC Incubation.** Bar plots display the mean ± SEM for three PI-defined populations (Δ 4hr – 0hr, %) across seven treatment conditions: unsorted control, pNCM, BTS, and LTX media, with each sorted treatment supplemented with either 1% BSA or 0.1% PVA. Membrane integrity was assessed using propidium iodide (PI) fluorescence via Image-Based Flow Cytometry, and populations were categorized as: (a) PI Negative (intact membrane), (b) PI Transitioning (partial compromise), and (c) PI Positive (membrane-compromised). Bars represent the change in frequency of each population from 0 hr to 4 hr incubation. Bars without letters indicate no significant differences.

**Supplementary Figure 7. Evaluation of Acrosomal Remodeling in Spermatozoa Using PNA-AF594 Fluorescence Across Treatments and Timepoints.** Bar plots display the mean ± SEM of sperm populations stained with PNA-AF594 at 0 hr (solid bars) and 4 hr (striped bars) across seven treatment groups: unsorted and pNCM, BTS, and LTX supplemented with 1% BSA or 0.1% PVA. Lectin peanut agglutinin (PNA) conjugated to AlexaFluor 594 was used to assess acrosomal integrity, with sperm classified as (a) PNA Negative (intact acrosome), (b) PNA Positive (remodeled/compromised acrosome), and (c) PNA Positive–Positive (extensive or advanced acrosomal remodeling/compromised). Bars without letters indicate no significant differences.

**Supplementary Figure 8. Change in Acrosomal Status of Spermatozoa Across Treatment Groups Following 4-Hour IVC Incubation.**
Bar plots display the mean ± SEM for three PNA-AF594-defined populations (Δ 4hr – 0hr, %) across seven treatment conditions: unsorted control, pNCM, BTS, and LTX media, with each sorted treatment supplemented with either 1% BSA or 0.1% PVA. Acrosomal membrane status was assessed by Image-Based Flow Cytometry using peanut agglutinin (PNA) conjugated to Alexa Fluor 594, with populations defined as: (a) PNA Negative (intact acrosome), (b) PNA Positive (partial acrosome reaction), and (c) PNA Positive-Positive (complete acrosome reaction). Bars represent the change in frequency of each population from 0 hr to 4 hr incubation. Bars without letters indicate no significant differences.

**Supplementary Figure 9. Change in Mitochondrial Membrane Potential of Spermatozoa Across Treatment Groups Following 4-Hour IVC Incubation.** Bar plots display the mean ± SEM for three MitoTracker Deep Red-defined populations (Δ 4hr – 0hr, %) across seven treatment conditions: unsorted control, pNCM, BTS, and LTX media, with each sorted treatment supplemented with either 1% BSA or 0.1% PVA. Mitochondrial membrane potential was assessed using MitoTracker Deep Red via Image-Based Flow Cytometry, with populations defined as: (a) MitoTracker Negative, (b) MitoTracker Transitioning, and (c) MitoTracker Positive. Bars represent the change in frequency of each population from 0 hr to 4 hr incubation. Bars without letters indicate no significant differences among treatments.
